# Mucosal Bacterial Immunotherapy Attenuates the Development of Experimental Colitis by Reducing Inflammation Through the Regulation of Myeloid Cells

**DOI:** 10.3390/ijms252413629

**Published:** 2024-12-20

**Authors:** Eva Jiménez, Alberto Vázquez, Sara González, Rosa Sacedón, Lidia M. Fernández-Sevilla, Alberto Varas, Jose L. Subiza, Jaris Valencia, Ángeles Vicente

**Affiliations:** 1 Department of Cell Biology, Faculty of Medicine, UCM, 28040 Madrid, Spain; evajp@ucm.es (E.J.); alvazq02@ucm.es (A.V.); sagonz25@ucm.es (S.G.); rmsacedo@ucm.es (R.S.); avaras@ucm.es (A.V.); 2 Health Research Institute of the Hospital Clínico San Carlos (IdISSC), 28040 Madrid, Spain; lidia.martinez@urjc.es; 3 Health Research Institute of the Hospital Doce de Octubre (i+12), 28041 Madrid, Spain; 4 Department of Basic Health Sciences, Faculty of Health Sciences, University Rey Juan Carlos, 28922 Alcorcón, Spain; 5 Inmunotek S.L., Alcalá de Henares, 28805 Madrid, Spain; jlsubiza@inmunotek.com

**Keywords:** ulcerative colitis, inflammation, bacterial immunotherapy, vaccine, MV130, immunomodulation, macrophages, neutrophils

## Abstract

Ulcerative colitis is a chronic relapsing–remitting and potentially progressive form of inflammatory bowel disease in which there is extensive inflammation and mucosal damage in the colon and rectum as a result of an abnormal immune response. MV130 is a mucosal-trained immunity-based vaccine used to prevent respiratory tract infections in various clinical settings. Additionally, MV130 may induce innate immune cells that acquire anti-inflammatory properties and promote tolerance, which could have important implications for chronic inflammatory diseases such as ulcerative colitis. This work demonstrated that the prophylactic administration of MV130 substantially mitigated colitis in a mouse model of acute colitis induced by dextran sulphate sodium. MV130 downregulated systemic and local inflammatory responses, maintained the integrity of the intestinal barrier by preserving the enterocyte layer and goblet cells, and reduced the oedema and fibrosis characteristic of the disease. Mechanistically, MV130 significantly reduced the infiltration of neutrophils and pro-inflammatory macrophages in the intestinal wall of the diseased animals and favoured the appearance of M2-polarised macrophages. These results suggest that MV130 might have therapeutic potential for the treatment of ulcerative colitis, reducing the risk of relapse and the progression of disease.

## 1. Introduction

Ulcerative colitis (UC) is an inflammatory bowel disease (IBD) affecting the colon and rectum that has a markedly detrimental impact on patients’ quality of life, increasing the risk of colectomy and colon cancer. UC has a global prevalence of 5 million cases, with an increasing incidence in recent decades [1,2]. Although the prevalence of UC is similar in men and women, the symptoms of the disease are more pronounced in the latter [3]. UC is characterised by prolonged inflammation and superficial ulcerative disease of the colon. The underlying aetiology remains unclear but involves a complex interplay of genetic, environmental, and immunological factors that compromise the intestinal epithelial barrier, allowing luminal antigens to interact with the underlying immune system and trigger an inappropriate immune response [2,4]. This aberrant immune response is associated with the dysregulation of both adaptive and innate immunity, and also involves non-immune cells such as epithelial cells or cells of the lamina propria. In this regard, UC is primarily characterised by an increased abundance and activation of macrophages and neutrophils [4]. The former produce high levels of pro-inflammatory cytokines [5,6]. Neutrophils exhibit a dual role in UC and, following excessive recruitment and activation, are also important mediators of the activation of other immune cells, tissue damage, and the disruption of the intestinal epithelial mucosal barrier [4,7,8,9].

UC patients are initially treated with anti-inflammatory drugs, such as mesalazine (5-ASA) or corticosteroids. Maintenance therapy is based on the use of immunosuppressants (e.g., thiopurines), biologics (e.g., anti-TNF, anti-IL-12, anti-IL-23, or anti-integrins), and small molecules (e.g., Janus kinase inhibitors and sphingosine-1 receptor modulators) [2,10,11]. A number of new targets are now in clinical trials [12]. Other therapeutic strategies under investigation are based on restoring the gut microbiota, including faecal microbiota transplantation, which appears to improve clinical and endoscopic remission rates in UC patients compared to untreated patients, according to a recently published meta-analysis [13,14]. Despite these advances, remission rates remain limited, ranging between 30 and 60% across different patient populations. Lack of response, relapse, and side effects are constantly reviewed and treatments are appropriately modified [2]. In this context, an increasing number of studies are focusing on the search for new strategies to regulate the harmful chronic inflammatory response that accompanies UC and that ultimately is responsible for the main symptoms of the disease and the greater predisposition that UC patients have to develop colorectal cancer. 

MV130 is a mucosal polybacterial vaccine consisting of inactivated whole-cell bacteria of selected strains. MV130 has been described as a trained immunity-based vaccine (TIbV) conferring protection against respiratory viruses and preventing recurrent respiratory infections [15,16,17,18,19], including in immunosuppressed patients [20,21,22,23]. MV130 primes human dendritic cells (DCs) to promote Th1/Th17 and IL-10 immune responses [24]. MV130 has been demonstrated to induce the reprogramming of both murine bone marrow progenitor cells and human monocytes in vitro, resulting in an increased secretion of cytokines, including TNFα, IFNγ, IL-1, and IL-6 [15]. Nevertheless, trained innate immune cells with specific microbial stimuli may also show anti-inflammatory characteristics and promote tolerance [17]. Interestingly, our group has shown that MV130 also modulates the functionality of the mesenchymal stem cells (MSCs) residing in the oral mucosa [25]. These cells are able to capture, process, and retain Toll-like receptor (TLR) ligands derived from MV130 digestion. Furthermore, in an in vivo model of LPS-induced acute inflammation, MV130-pretreated MSCs are more efficient in resolving the inflammatory process, primarily by regulating leukocyte local infiltration and promoting the generation of macrophages and DCs with immunosuppressive properties [25]. MV130 may therefore exert a direct regulatory effect on cell populations involved in the pathogenesis of UC, suggesting its potential as a protective agent against this disease.

The aim of the present study was to evaluate whether preventive treatment with MV130 could improve the course of UC by reducing inflammation, as has been observed in other pathological conditions. Our findings indicate that, in a dextran sulphate sodium (DSS) induction animal model, MV130 treatment is able to ameliorate disease symptoms by reducing systemic inflammation and intestinal mucosa damage through the modulation of the gut macrophage and neutrophil subpopulations. 

## 2. Results

### 2.1. MV130 Attenuates Disease Progression and Reduces the Main Anatomopathological Signs of Ulcerative Colitis

**Figure 1:**
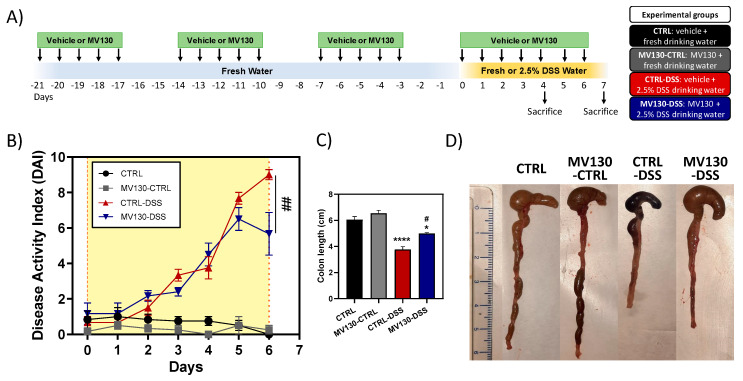
Beneficial effect of prophylactic MV130 treatment on the development of acute colitis. (**A**) Schematic diagram of study design. Briefly, after 21 days of prophylactic MV130 treatment or vehicle, acute colitis was induced in the animals by treatment with 2.5% DSS for 7 days. (**B**) The disease activity index (DAI) was calculated as specified in the Materials and Methods and the mean value ± SEM for each day is plotted in the graph. Animals fed fresh water were used as controls. (**C**) The mean colon length ± SEM of 4 animals per group is shown. (**D**) Representative macroscopic image of the colon from one animal in each group. Graph shows the mean values ± SEM of the different groups of animals. Significance is indicated with respect to CTRL (*) or CTRL-DSS (#) (* *p* < 0.05; **** *p* ≤ 0.0001; # *p* < 0.05 and ## *p* < 0.01)—analysed by Kruskal–Wallis test for DAI and one-way ANOVA for colon length).

We aimed to determine whether MV130 could exert a protective effect in the development of UC. To this end, after 21 days of prophylactic treatment with the drug (MV130 animals) or vehicle (CTRL animals), the disease was induced by the administration of DSS in drinking water (DSS animals), while mice given fresh water (CTRL animals) were used as controls (Figure 1A). As previously described, the induction of acute UC by DSS resulted in a progressive increase in the disease activity index (DAI), characterised by bloody diarrhoea and sustained weight loss [26,27]. Therefore, the DAI of the different groups of mice was initially determined. A progressive increase in disease activity was observed from day 2 in the DSS-treated animals. In contrast, animals previously treated with MV130 had a lower increase in DAI from day 5, with a clear improvement in disease severity on day 6, when disease symptoms are most critical. This resulted in significant differences between the two groups of animals in which the disease was induced (CTRL-DSS and MV130-DSS). It is noteworthy that treatment with MV130 itself (MV130-CTRL group) had no effect on the weight or consistency of the faeces of the animals and did not modify the DAI parameters in comparison to the control group (CTRL group; Figure 1B).

The anatomopathological study revealed that the colon of CTRL-DSS mice exhibited a significant shortening (Figure 1C,D) similar to that previously described by other groups [27]. In animals previously treated with MV130 (MV130-DSS), the shortening was significantly less pronounced (Figure 1C,D), with these effects being evident as early as day 4 after DSS induction and reaching statistical significance at the endpoint of the study (day 7) (Figure 1C,D). 

### 2.2. MV130 Modulates Systemic Pro-Inflammatory Mediators and Myeloid Cell Populations in Spleen and Mesenteric Lymph Nodes During Acute Colitis

**Figure 2:**
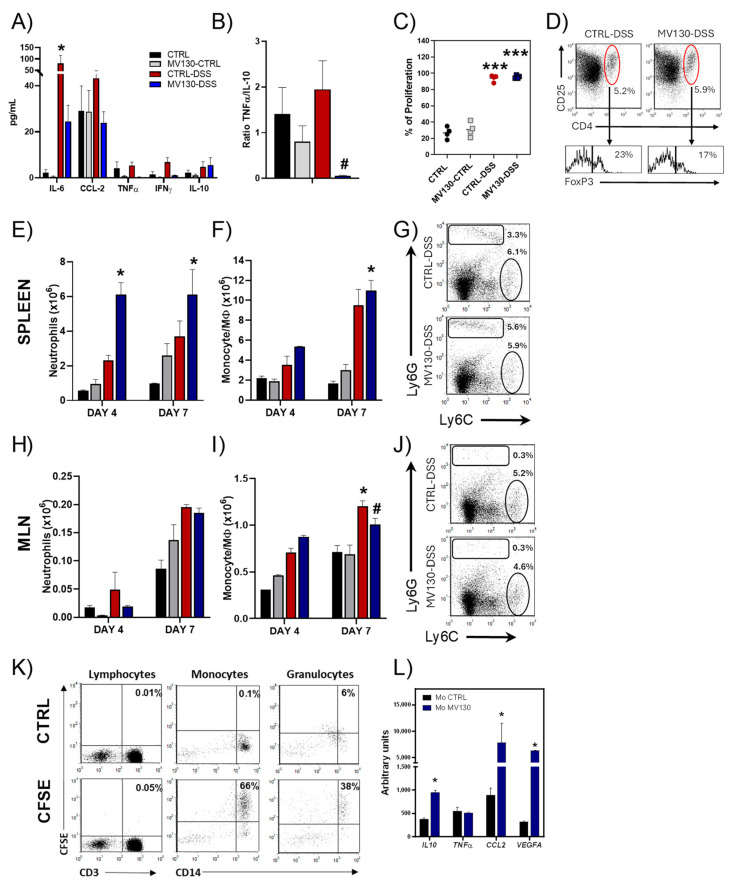
Systemic effects of MV130 in colitis-induced animals. (**A**–**J**) Mice were treated as described in Figure 1A, and samples were collected on day 4 and 7 after colitis induction. (**A**) Serum levels of several cytokines in the different groups of mice 7 days after DSS treatment (n = 4 per group) were measured by CBA. (**B**) Data represent TNFα/IL-10 ratio production in each experimental group. (**C**) Splenocytes were labelled with CFSE and stimulated with ConA + PMA for four days, as described in the Material and Methods. The percentage of proliferation of splenocytes from mice of different experimental groups is shown (n = 4 per group). Flow cytometry analysis of spleen (**D**–**G**) and mesenteric lymph node immune populations (**H**–**J**). (**D**) Representative dot plots of the Treg lymphocyte population (CD4^+^CD25^+^FoxP3^+^) in the spleen after four days of stimulation with ConA and PMA in CTRL-DSS and MV130-DSS mice (n = 4 per group). Absolute numbers of neutrophils (**E**,**H**) and monocytes (**F**,**I**) recovered from the spleen and MLN of mice on days 4 and 7 after colitis induction (n = 3–4 per group). (**G**,**J**) Representative dot plot of myeloid populations on day 7 after DSS induction. Neutrophils (Ly6G^hi^Ly6C^lo/−^) and monocyte/macrophage populations (Ly6G^−^Ly6C^+^) were gated within CD45^+^ CD11b^+^. (**K**) Flow cytometry analysis of human PBMCs after culture with MV130-CFSE for 24 h. Dot plots show the percentage of uptake of CFSE-MV130 by lymphocytes, monocytes, and granulocytes. Data are representative of 3 independent experiments. (**L**) mRNA expression of several cytokines was studied in monocytes treated with MV130 for 24 h by RT-qPCR. The graphs show the mean values ± SEM and the significance is indicated with respect to CTRL (*) or CTRL-DSS (#). * *p* < 0.05; *** *p* < 0.001; # *p* < 0.05—analysed by (**A**,**B**,**E**,**F**,**H**,**I**) Kruskal–Wallis, (**C**) one-way ANOVA, and (**L**) Mann–Whitney test.

The clear beneficial effect of MV130 on the clinical course of colitis motivated us to further investigate the systemic effect of MV130 treatment on the diseased animals. As expected, in correlation with the deterioration of the animals, we observed that circulating levels of pro-inflammatory proteins such as CCL2, TNFα IFNγ, and IL-6 were increased in the DSS control group compared to the healthy controls (Figure 2A). The levels of these pro-inflammatory mediators were lower in MV130-DSS mice, in many cases with mean levels similar to those seen in the healthy controls. In contrast, the levels of the anti-inflammatory cytokine IL-10, which appeared slightly increased at the end of the disease, were similar in the CTRL-DSS and MV130-DSS animals (Figure 2A). Thus, the TNFα/IL-10 ratio, as a measure of the level of systemic inflammation, was significantly reduced in UC animals previously treated with MV130 (Figure 2B).

Both the CTRL-DSS and MV130-DSS animals presented an evident splenomegaly as early as 4 days after the induction with DSS, becoming statistically significant on day 7 when a generalised increase in total splenocytes and CD4^+^ T lymphocytes was observed in both groups of animals (Supplementary Figure S1A,B). In correlation with these results, splenic T cells from DSS-treated animals showed a higher proliferative response to ConA + PMA than splenocytes from the CTRL or MV130-CTRL animals, with no significant differences between the CTRL-DSS and MV130-DSS groups (Figure 2C). Although more modest than in the spleen, 7 days after disease induction, the number of cells in the MLN of CTRL-DSS mice also increased, and no differences were observed in MV130-treated mice (Supplementary Figure S1C). On the contrary, MV130 treatment significantly reduced the increase in CD4^+^ T lymphocytes present in the lymph nodes of diseased animals (Supplementary Figure S1D). 

Since CD4^+^CD25^+^FoxP3^+^ Treg lymphocytes have been described to play an important role in the development and control of inflammatory bowel disease, we next analysed the representation of this population in the spleen and the MLNs of the different groups of animals after in vitro stimulation. As shown in Figure 2D and Figure S1E, no differences were observed in the numbers of Treg cells after MV130 treatment, suggesting that the effect of drug treatment on ameliorating disease symptoms was not due to an increase in this cell population. 

Similar to the results described by other authors [28,29], we observed that colitis induction tended to increase the myeloid populations of both neutrophils (CD11b^+^Ly6G^hi^Ly6C^lo/−^) and monocyte/macrophages (Mϕ) (CD11b^+^Ly6G^−^Ly6C^+^). However, MV130-DSS animals presented an even greater increase in neutrophil numbers as early as 4 days after disease induction and in the monocyte/Mϕ population at day 7, compared to healthy animals (Figure 2E–G). Moreover, our results showed that the monocyte/Mϕ population was increased in the MLNs of CTRL-DSS mice and that MV130 treatment was able to significantly attenuate this increase (Figure 2H–J). These results suggested that myeloid populations may be the target cells of MV130 in DSS-induced colitis. Supporting this idea, in vitro studies indicated that both neutrophils and mainly monocytes from human PBMCs efficiently capture MV130-CFSE, unlike lymphocytes, which did not show any labelling in the flow cytometry analyses (Figure 2K). In addition, isolated human monocytes treated with MV130 showed a significantly increased expression of *IL-10*,* CCL2*, and *VEGF*, with no changes in the production of *TNFα* (Figure 2L).

### 2.3. MV130 Preserves Colonic Cytoarchitecture, Reducing Leukocyte Infiltration, Fibrosis, and Oedema

**Figure 3:**
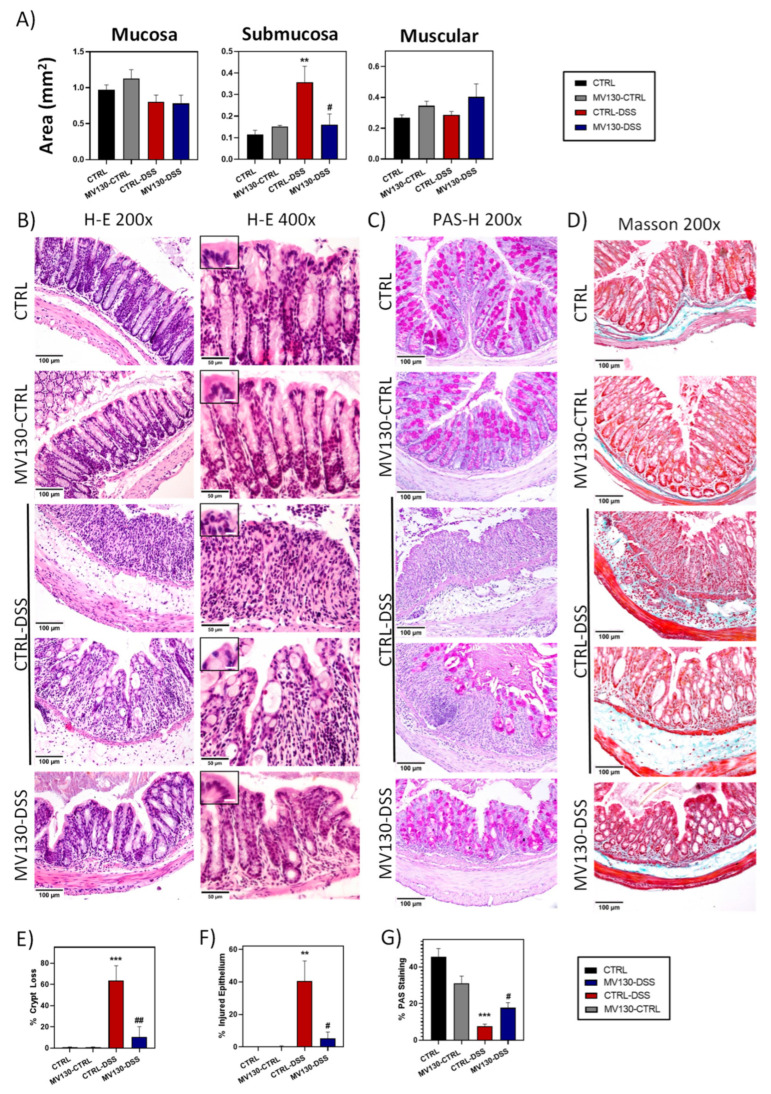
MV130 treatment prevents colitis-induced tissue damage. (**A**) Haematoxylin and eosin staining was performed, and the areas of the different histological regions were measured. Graphs show the mean values ± SEM of the different groups of animals. Representative images of colon sections from each group of animals: (**B**) haematoxylin and eosin (scale bar: left column, 100 μm; right column, 50 μm; insert, 10 μm); (**C**) PAS–haematoxylin (scale bar, 100 μm); and (**D**) Masson’s trichrome (scale bar, 100 μm). (**E**) Percentage of area with loss of crypt cytoarchitecture in the mucosa. (**F**) Percentage of injured epithelium. (**G**) Quantification of goblet cells in the mucosa using PAS staining. Graphs represent mean ± SEM. The significance is indicated with respect to CTRL (*) or CTRL-DSS (#) (** *p* < 0.01; *** *p* < 0.001; # *p* < 0.05; ## *p* < 0.01—analysed by one-way ANOVA test).

The histopathological analysis of colon sections revealed that, while colonic tissue from the CTRL-DSS animals showed an evident enlarged submucosa, prophylactic treatment with MV130 resulted in a statistically significant control of the thickening, with the submucosal area in these MV130-DSS animals being similar to that of the healthy controls (Figure 3A).

CTRL-DSS mice exhibited a significant histopathological alteration in the colonic mucosa and submucosa in comparison to healthy animals. Although in some areas the colonic crypts were partially visible, in most of the mucosa, the characteristic intestinal structures were not observable, and the entire tissue was extensively infiltrated by leukocytes (Figure 3B, right column). However, in MV130-DSS animals, the crypts were only slightly disorganised, and the cytoarchitecture of the colon was mostly preserved throughout the section. Most of the damage described in untreated DSS diseased animals was not observed in the MV130-DSS group, and only minor areas of infiltration were present (Figure 3B). In addition, the prophylactic treatment of the disease-induced animals with MV130 was able to almost completely prevent epithelial damage, showing columnar enterocytes instead of the flattened morphology characteristic of the intestinal epithelium of the CTRL-DSS mice (Figure 3B). The quantification of tissue damage revealed that over 60% of the crypt area was altered in DSS-treated animals, and 40% of the epithelial surface had a completely flattened appearance (Figure 3E,F). In contrast, significantly lower percentages were found in animals pretreated with MV130, with 11% and 8% damage, respectively, in these entities (Figure 3E,F).

Importantly, the histological examination of intestinal sections from CTRL-MV130 animals revealed that the drug did not induce any alterations to the tissue architecture when compared with CTRL mice (Figure 3).

The next objective was to ascertain whether the disappearance of the crypt structure was accompanied by changes in the goblet cells. To this end, a study was carried out using PAS–haematoxylin staining and the quantification of the percentage of the PAS-labelled area as a measure of the amount of mucin in the mucosa. The administration of MV130 in the control animals resulted in a slight, non-significant reduction in PAS labelling, mainly in the deep zone of the crypts (Figure 3C,G). However, in DSS mice, goblet cells were absent in areas of damaged crypts and epithelia, and their numbers were reduced in areas where crypts were slightly preserved (Figure 3C,G). Furthermore, a substantial release of the secretion product of these cells into the lumen of the colon was observed after 7 days of colitis development (Figure 3C). In colitis-induced animals treated with MV130, PAS-positive cells did not reach the values of control animals, but goblet cell preservation was significant compared to DSS mice (Figure 3C,G). 

Finally, while Masson’s trichrome staining revealed intestinal fibrosis, oedema, and cellular infiltration in the colonic submucosa of CTRL-DSS animals, pre-treatment with MV130 prevented the appearance of these inflammatory manifestations in the MV130-DSS group (Figure 3D). 

Overall, the histological structure of the colons of DSS mice treated with MV130 was notably better preserved, supporting a good therapeutic outcome.

### 2.4. MV130 Attenuates Colonic Leukocyte Infiltration and Remodels Macrophage Phenotype and Ratio

**Figure 4:**
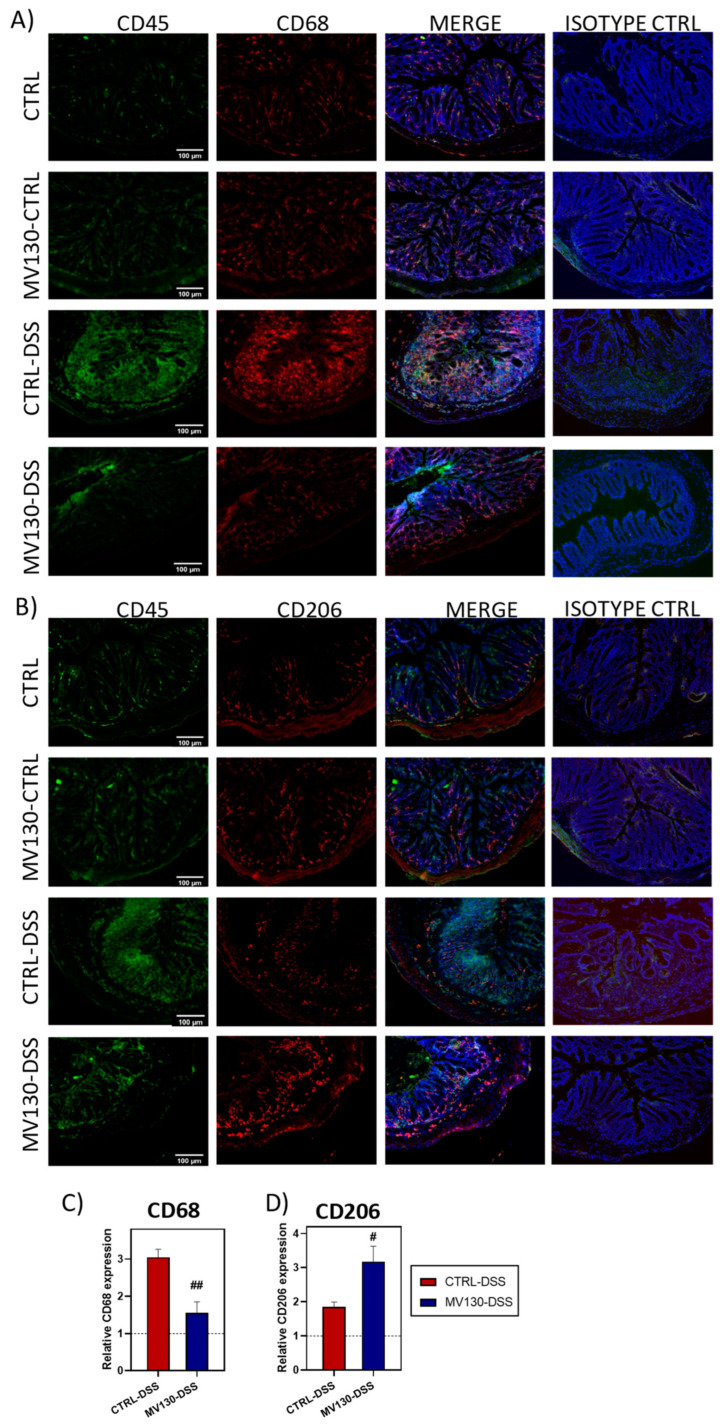
MV130 treatment reduces innate immune cell infiltration and modulates the macrophage phenotype. Colon cryosections from the different experimental groups of mice sacrificed on day 7 were immunostained for CD45 (green) and for (**A**) CD68 (red) or (**B**) CD206 (red). The isotype controls are also shown. Hoechst was used for nuclear counterstaining. Scale bars represent 100 mm. Images are representative of four animals per group. Measurement of (**C**) CD68^+^ and (**D**) CD206^+^ macrophages in the colonic mucosa and submucosa of mice from the different groups was performed. Relative CD68 or CD206 expression was calculated by dividing all individual data by the mean expression in the CTRL group of animals. Results represent the mean ± SEM of four different animals, and the significance is indicated with respect to CTRL-DSS (# *p* < 0.05; ## *p* < 0.01—analysed by Student’s *t* test).

Confirming the histological results shown above, which indicate that leucocyte infiltration in the colons of disease-induced animals was reduced by preventive treatment with MV130, the immunofluorescence study showed that while CTRL-DSS animals exhibited a notable infiltration of CD45^+^ cells within the colon lamina propria and submucosa, this infiltration was prevented in MV130-DSS mice (Figure 4A,B). We therefore set out to extend our research by investigating the myeloid populations that play a central role in the inflammatory process and infiltrate the colon wall, focusing initially on the possible effects of MV130 treatment on macrophage subpopulations. As shown in the immunofluorescence images, the development of disease in the CTRL-DSS group was accompanied by a significant increase in pro-inflammatory CD68^+^ M1-like macrophages in the colonic mucosa and submucosa. However, in the MV130 group, disease induction did not result in such recruitment of inflammatory macrophages, as shown by CD68 labelling comparable to that observed in the healthy control group (Figure 4A). On the contrary, a greater infiltration of immunomodulatory CD206^+^ macrophages (M2-like macrophages) was observed in the mucosa, particularly in the lamina propria of MV130-DSS mice, compared to both the healthy and untreated diseased animals (Figure 4B). It should be noted that the administration of MV130 to healthy animals did not significantly modify the distribution or number of colonic macrophages; thus, treatment with MV130 only produced the changes described in the macrophage populations when the inflammatory process was subsequently induced by DSS. 

Therefore, prophylactic treatment with MV130 in diseased animals reduced the presence of pro-inflammatory CD68^+^ macrophages while increasing that of immunosuppressive CD206^+^ macrophages, as demonstrated by the quantification of both populations in the colon (Figure 4C,D).

### 2.5. MV130 Switches Macrophage Differentiation Towards an M2-like Anti-Inflammatory Phenotype

**Figure 5:**
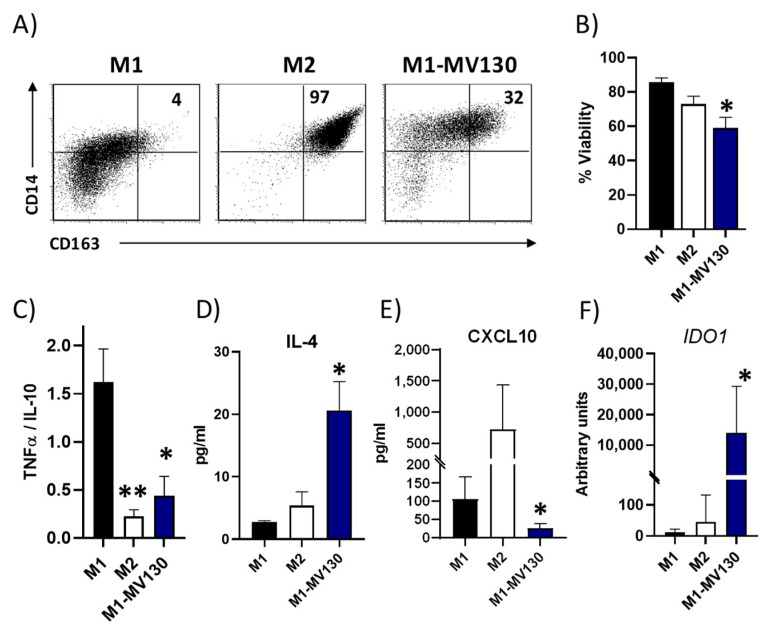
MV130 induces M2-like macrophage differentiation. Human CD14^+^ monocytes were cultured for 6 days with GM-CSF to induce M1 macrophages or with M-CSF to induce M2 macrophages. MV130 treatment was added at the beginning of the culture. (**A**) Dot plots show the percentage of M2-like CD14^+^CD163^+^ cells present in the different culture conditions (representative of 4 independent experiments). (**B**) Percentage of macrophage viability was measured by flow cytometry. Annexin V^−^ PI^−^ cells were considered as viable cells. (**C**–**E**) After 6 days of culture, supernatants were analysed for different immunomodulatory factors produced by macrophages. (**C**) Data represent TNFα/IL-10 ratio production, (**D**) IL-4, and (**E**) CXCL10 protein secretion by macrophages under the different experimental conditions. (**F**) *IDO1* mRNA expression levels quantified in macrophages under the different experimental conditions at day 6 of culture. (**B**–**F**) Data are mean ± SEM of four independent experiments. Significance is indicated relative to M1 control (* *p* < 0.05; ** *p* < 0.01—analysed by Kruskal–Wallis test).

We next studied whether MV130 promotes the in vitro repolarization of human pro-inflammatory M1 macrophages toward an anti-inflammatory M2 phenotype. As shown in Figure 5A, the population of M1 macrophages differentiated in the presence of MV130 (M1-MV130) included an increased proportion of M2-like CD14^+^CD163^+^ cells. Interestingly, MV130 also reduced the viability of M1 cells (Figure 5B). In addition, the ratio of pro-inflammatory to anti-inflammatory cytokines (TNFα/IL-10) produced by M1-MV130 macrophages was significantly lower than that produced by control M1 macrophages, and an increase in IL-4 production was also observed (Figure 5C,D). M1 macrophages, differentiated in the presence of MV130, also exhibited a significantly reduced secretion of the chemokine CXCL10, whose role as a chemoattractant of Th1 cells is well known [27] (Figure 5E). Additionally, *IDO* mRNA expression was markedly increased in M1-MV130 macrophages (Figure 5F), suggesting that MV130 redirects the differentiation of M1 macrophages to an M2-like phenotype.

### 2.6. MV130 Reduces the Recruitment of Neutrophils and Their Pro-Inflammatory Mediators in the Colon of Animals with Ulcerative Colitis

**Figure 6:**
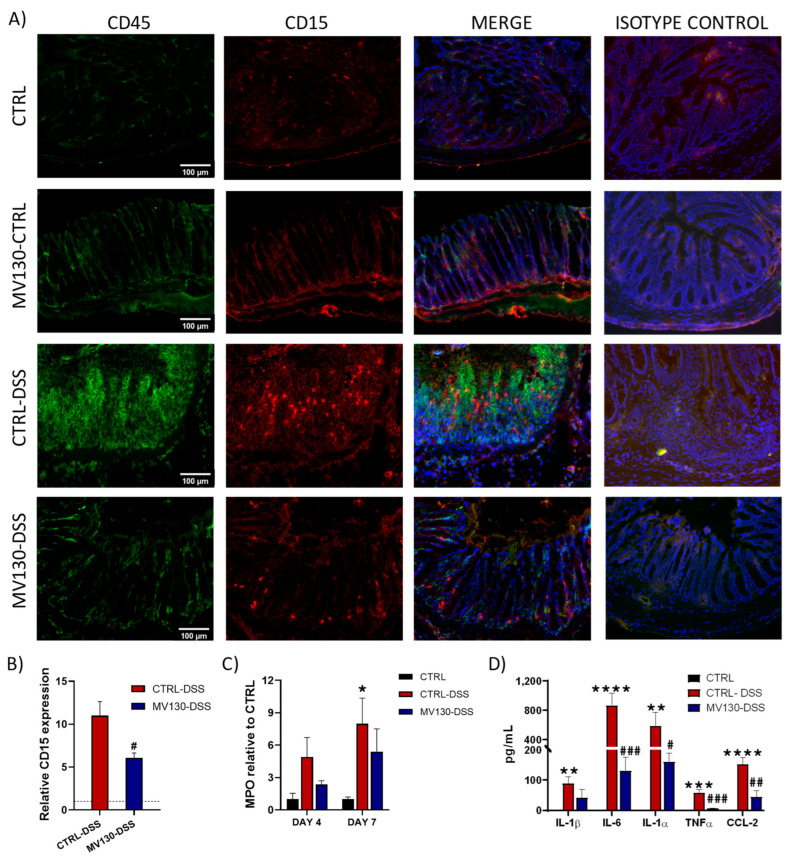
MV130 reduces the recruitment of neutrophils and tissue damage mediators in ulcerative colitis. (**A**) Immunodetection of CD45 (green) and CD15 (neutrophils, red) in colon cryosection 7 days after DSS induction. The isotype controls are also shown. Hoechst was used for nuclear counterstaining. Scale bars represent 100 μm. Images are representative of four animals per group. (**B**) Measurement of CD15^+^ neutrophils in the colonic mucosa and submucosa of mice from the different groups was performed (dotted line). Relative CD15 expression was calculated by dividing all individual data by the mean expression in the CTRL group. (**C**) Relative MPO activity in colonic protein extracts on days 4 and 7 after colitis induction. (**D**) Protein levels in colonic mucosa measured by flow cytometry. (**B**–**D**) Results represent the mean ± SEM of four different animals per group and the significance is indicated with respect to CTRL (*) or CTRL-DSS (#). * *p* < 0.05; ** *p* < 0.01; *** *p* < 0.001; **** *p* < 0.0001; # *p* < 0.05; ## *p* < 0.01; ### *p* < 0.001—analysed by (**B**) Student’s *t* test, (**C**) Kruskal–Wallis test, (**D**) and one-way ANOVA test.

As shown in Figure 6A, the development of UC was accompanied by a marked recruitment of CD15^+^ granulocytes preferentially located in the middle and upper regions of the colonic crypts. In contrast, in diseased animals pre-treated with MV130 (MV130-DSS), the number of CD15^+^ cells present in the colon was drastically reduced, with their presence mainly confined to the lower mucosa (Figure 6A). The quantification of CD15 expression showed that although the number of neutrophils in MV130-DSS animals was higher than in healthy controls, it was significantly lower than in CTRL-DSS animals (Figure 6B). However, in animals in which the disease was not subsequently induced, MV130 treatment had no effect on CD15^+^ cell numbers, and the immunofluorescence images were similar to those of the healthy controls (Figure 6A).

Consistent with a lower neutrophil influx in MV130-DSS animals, decreased levels of myeloperoxidase (MPO) were detected in their colon tissue compared to CTRL-DSS animals (Figure 6C). This was also observed for pro-inflammatory soluble mediators such as IL-1α, IL-6, IL-1β, TNF-α, and CCL2 (Figure 6D). 

Taken together, these results suggest that pre-treatment with MV130 can attenuate the colon inflammatory response associated with DSS-induced colitis.

## 3. Discussion

The therapeutic capacity of MV130 for recurrent infections and inflammatory processes has previously been pointed out [16,17,19,21,22,25,30,31], showing a broad range of both non-specific and specific immune responses in mucosal and extra-mucosal tissues [32,33,34]. Here, we provide experimental evidence in a DSS-induced colitis mouse model showing that pre-treatment with MV130 significantly improves clinical and histological scores compared to untreated DSS animals. The observed therapeutic effect of MV130 in the colitis model could be attributed to its capacity to control the local inflammatory process, likely reflecting the systemic activity of MV130.

At the systemic level, MV130-DSS animals showed splenomegaly, with a significant increase in myeloid CD11b^+^Ly6G^hi^Ly6C^−/low^ cells. This cell population could be responsible, at least in part, for the reduction in the progression of the disease, as has already been described by Zhang et al. [29]. These authors observed that the transplantation of splenic Gr1^+^CD11b^+^ cells to DSS-treated mice during the early phase of disease induction reduced inflammation and promoted efficient colonic mucosal healing [29]. Other authors have also described that during the induction of chronic inflammation, there is a recruitment of myeloid cells with suppressive potential from the bone marrow to the spleen. Moreover, these splenic CD11b^+^Ly6G^high^ myeloid cells play an important role in the induction of the immunosuppressive environment [35]. Whether the population accumulated in the spleen of MV130-DSS mice has immunosuppressive properties requires further study. On the other hand, although the participation of lymphocyte populations in the control of this inflammatory response cannot be ruled out, the study of Treg lymphocytes in the spleen and MLNs showed no increase in the treated animals. This suggests that Treg cell population would not contribute, or contribute minimally, to the observed therapeutic effects of MV130. In any case, further studies aimed at analysing the T-lymphocyte populations present in the colon are necessary to clarify this issue.

Histological analysis of the colon demonstrated that, consistent with other studies [27,36], DSS treatment results in significant mucosal disruption, characterised by extensive crypt loss, notable damage to the epithelial monolayer, and the loss of goblet cells. This is accompanied by a considerable infiltration of leukocytes into the lamina propria, extending down to the submucosa. While the mechanism behind the disruption of the mucosa and the epithelial barrier remains largely unknown [4], it is believed that the disease is perpetuated by the unsuccessful resolution of inflammation [37]. It is also unclear whether the breakdown of the epithelial barrier precedes or follows the development of inflammation in the lamina propria [38]. Our results show that MV130 treatment can maintain the integrity of the entire mucosal cytoarchitecture and prevent tissue damage. The role of MV130 in controlling inflammation may have an indirect impact on preserving the epithelial barrier, since it could reduce the infiltration of cells such as neutrophils and macrophages, as discussed below. While these cells are capable of controlling the spread of microorganisms, they also secrete mediators that impair the epithelial barrier, leading to cell death, the disruption of epithelial cell junctions [4,39], and the depletion of goblet cells [4,40].

Alternatively, it is possible that MV130 exerts a direct effect on epithelial cells via TLRs. This signalling plays a pivotal role in maintaining a healthy epithelial barrier; regulating the proliferation, apoptosis, and differentiation of crypt cells; promoting goblet cell differentiation and mucus secretion; and enhancing antimicrobial responses that can control dysbiosis [41]. Furthermore, once inflammation takes place, TLR signals can accelerate epithelial repair and, accordingly, several studies have evaluated the use of TLR ligands as therapeutic tools to ameliorate colitis and accelerate recovery [41,42]. Based on these observations, TLR signalling triggered by MV130 could prevent epithelial damage, as well as the loss of goblet cells and their mucus layer in animals treated with DSS. It can also be hypothesised that the bacterial strains in MV130 could specifically provide balanced signals to epithelial cells, maintaining their homeostatic function and preventing DSS-induced damage. Numerous studies have suggested the linkage of gut microbiota to inflammatory diseases, including UC. It has been demonstrated that the abundance of multiple orders, classes, families, genera, and species of bacteria that make up the intestinal microbiota differs between patients with inflammatory bowel disease and control individuals, and it has been suggested that this relates to colorectal cancer development [13,43]. Maintaining the integrity of the epithelial barrier along with the control of local inflammation, as observed in UC animals treated with MV130, could preserve the intestinal bacterial community, preventing or controlling the dysbiosis characteristics of UC patients and, ultimately, influencing the likelihood of colorectal cancer in these patients [13,44]. 

It has been described that in UC flares [45], as in acute DDS-induced colitis [46,47], contact between epithelial cells and pathogenic microorganisms in the colon activates the inflammasome. This activation leads to the hyperstimulation of mucus secretion, the death of exhausted sentinel goblet cells, and the subsequent attenuation of mucus secretion, triggering a vicious cycle of increased epithelial exposure to bacteria, an exacerbation of inflammation, and further breakdown of the mucosal barrier [48,49]. Other studies have revealed that treatment with TNF-α, one of the pro-inflammatory cytokines that is elevated in the colon of DSS animals, reduces the number of goblet cells [45]. Therefore, the protective effect of MV130 on goblet cells could be indirect, mediated—as has been described in other treatments [46]—through control of the inflammatory response. Or, conversely, MV130 could have a direct effect on goblet cells. In this regard, previous research has shown that treatment with *Escherichia coli* or *Propionibacterium freudenreichii* improves DSS-induced colitis in mice and rats, restoring goblet cell numbers and stimulating the secretion of a structurally correct mucus with proper barrier functions [47]. 

When the intestinal epithelial barrier is damaged, neutrophils are rapidly recruited from microcirculation to the injured tissue through a series of chemotactic gradients of cytokines such as IL-1β, IL-6, and TNFα, and chemokines such as CCL8, CXCL10, and CCL2 [4]. These molecules can be produced by immune cells, such as macrophages and Th17 cells, and also by epithelial cells following damage [50]. Therefore, a reduction in the levels of these molecules, as occurs in MV130-DSS mice, would reduce the influx of neutrophils into damaged tissues. Neutrophil infiltration is associated with disease activity in UC [9]. Several studies have demonstrated increased levels of neutrophils in patients with active IBD. Moreover, increased neutrophil activity is known to occur in patients with IBD, being associated with the release of TNFα factor, which contributes to neutrophil activation. Our results show that serum TNFα levels are reduced in diseased animals treated with MV130, which may, in turn, lead to reduced neutrophil activity in these animals. In this regard, levels of MPO, a lysosomal protein found in neutrophils and used as a biomarker to assess disease status [51], are significantly higher in patients with active and severe IBD compared to those with quiescent IBD and healthy subjects [51]. Consistent with the reduction in neutrophil infiltration mentioned above, our results show that treatment with MV130 reduces MPO levels in the colon of DSS animals, suggesting that the treatment helps control disease progression and/or severity. 

The recruitment of monocytes and their differentiation into pro-inflammatory or anti-inflammatory macrophages has been described as a key factor in the development of UC [52,53]. During intestinal inflammation, large numbers of Ly6C^high^ inflammatory monocytes are recruited to damaged tissue through a CCL2 chemoattractant protein gradient [54]. It is well known that the inhibition of the recruitment of these cells to the inflamed bowel reduces the symptoms of DSS-induced colitis [55]. Additionally, IBD patients present increased numbers of pro-inflammatory macrophages that produce pro-inflammatory molecules, such as TNFα, IL1β, and IL-6 [56]. Our results demonstrate that colonic levels of CCL2, TNFα, IL1β, and IL-6 are significantly reduced in DSS animals treated with MV130, which is associated with reduced numbers of CD68^+^ pro-inflammatory macrophages. On the other hand, some authors have shown that, under certain conditions, macrophages can remodel their phenotype and switch from M1 to M2 in DSS-induced colitis mice, promoting anti-inflammatory therapeutic effects [57,58]. In this sense, MV130 redirects monocyte differentiation toward an M2-like phenotype, reducing the pro-inflammatory/anti-inflammatory TNFα/IL-10 cytokine ratio and the levels of the Th1 attracting chemokine CXCL10, as well as increasing the levels of tolerogenic factors such as IL-4 and IDO1. These results correlate with the increased presence of CD206^+^ M2-like macrophages in the colon of MV130-DSS mice in the in vivo model. Therefore, our findings indicate that the MV130 polybacterial preparation provided a microenvironment favourable for the repolarisation of M1 macrophages towards an anti-inflammatory phenotype functionally similar to M2. 

Taken together, MV130 reduces systemic and local inflammation as well as intestinal mucosal damage through a mechanism involving differential recruitment and the polarisation of myeloid cell populations. Therefore, prophylactic treatment with MV130 could be a promising approach to preventing the progression of UC by reducing clinical relapses.

Limitations of the study: 

A question not addressed in this study is whether MV130 treatment has the same therapeutic effects in controlling the course of UC in female mice. 

Further studies would be also needed to elucidate whether MV130 treatment is able to correct the dysbiosis characteristics of UC patients and the mechanism involved. 

## 4. Materials and Methods

### 4.1. Mice

In this study, 6–8-week-old male C57BL/6 mice (Charles River Laboratories, Barcelona, Spain) were housed in the animal facilities (Registration No. ES280790000086) of the Complutense University of Madrid (UCM, Madrid, Spain). All mice were housed for at least 1 week for acclimation prior to experimental use. Animals were provided with water and food ad libitum and with environmental enrichment materials. They were routinely screened for pathogens according to FELASA procedures, and Spanish and European regulations (Spanish RD 53/2013 and Law 6/2013, European Directive 2010/63/UE and Recommendation 2007/526/EC) were followed for the care of the animals and the conduct of the experiments. Symptoms of disease other than colitis or weight loss of more than 20% were considered as endpoint and exclusion criteria and checked daily. However, no animal had to be excluded or euthanised before the scheduled time. We used cervical dislocation to euthanize the animals. The procedures were approved by the UCM Animal Experimentation Ethical Committee and by the Division of Animal Protection of the Comunidad de Madrid (PROEX 208.0/21).

### 4.2. Study Design, MV130 Treatment, and Induction of Acute Ulcerative Colitis with Dextran Sulphate Sodium (DSS)

Mice were randomly divided into two groups using a computer-based random order generator. The different groups of animals, placed in separated cages, were treated sublingually with 25 μL of MV130 at 300 FTU (Formazin Turbidity Units a/mL) or vehicle, representing MV130 and CTRL, respectively (Inmunotek SL. Madrid, Spain). Treatment was administered for 21 days in cycles of 5 days, with 2 days of rest between cycles, as illustrated in Figure 1A. Subsequently, four animals from each group were selected randomly and placed in separate cages, and acute colitis was induced in DSS-treated animals by administering 2.5% dextran sulphate sodium (DSS, molecular weight 20,000 Da, Sigma-Aldrich, St Louis, MO,USA) in the drinking water ad libitum from day 0 to day 4 or 7, depending on the experiment, while the other four control animals (CTRL) from each group received fresh water. During the colitis induction period, the animals continued to receive MV130 or vehicle daily. Two different experiments were conducted, in which animals from each of the four experimental groups (CTRL, MV130-CTRL, CTRL-DSS, and MV130-DSS) were sacrificed at 4 or 7 days. To guarantee the blinding procedure was effective, different researchers were assigned to different tasks, from treatment administration to sample collection. Therefore, one investigator administered the treatment to the animals, and was the only individual aware of the treatment group allocation. This investigator provided a code for each animal to ensure the traceability of the samples. A second investigator was responsible for the euthanasia procedure, while a third investigator performed the surgical procedure. Finally, different researchers (excluding the investigator who coded the animals) conducted the studies described in this paper.

The severity of colitis was evaluated daily by scoring clinical disease activity on a scale of 0–11, assessing weight loss, stool consistency, and the presence of faecal blood. The three components were then summed to yield a total score: weight loss (0 = 0%, 1 = 5–10%, 2 = 11–15%, 3 = 16–20%), stool consistency (0 = normal stool, 2 = soft stool, 4 = diarrhoea), and presence of faecal blood (0 = absent, 0.5 = present but minimal, 1 = blood clearly detected, 2 = visible bleeding, 3 = gross bleeding and around the anus). Mice were sacrificed on day 4 or 7 depending on the experiment. The entire colon was removed, from the caecum to the anus, and the length of the colon was measured as an indirect marker of inflammation. The colons were then divided into three equivalent parts for the analyses described in the following sections.

At the time of sacrifice, blood was collected for serum protein analysis. Spleen and mesenteric lymph nodes (MLNs) were collected for a systemic analysis of lymphoid and myeloid subpopulations by flow cytometry.

The entire colon was removed and divided into three equal parts. The proximal part was always reserved for the analysis of myeloperoxidase activity, the middle third for the measurement of colonic proteins, and the distal third for histological examination.

### 4.3. Histological Analysis

The first part of the distal third of the colon was fixed in 4% paraformaldehyde, embedded in paraffin, and sliced into 4 µm sections. Following staining with haematoxylin and eosin (H&E), PAS–haematoxylin, or Masson’s trichrome, histological analysis was conducted in a blinded manner. The distinct histological regions of the colon, the injured epithelium or crypts, and the proportion of PAS-stained area of the entire sections were quantified using Fiji-win64 software (https://fiji.sc/, last access 2 October 2024).

### 4.4. Analysis of Serum Proteins

Serum samples were obtained from the blood of animals using BD SST Microtainer^®^ blood collection tubes, which contain an inert gel barrier and a clot activator coating, followed by centrifugation at 13,000 rpm for 3 min. Several cytokines were measured in serum using a Cytometric Bead Array (CBA) Mouse Inflammation kit (BD Biosciences, San José, CA, USA). The theoretical detection ranges were as follows (pg/mL): IL-6 (5.2–5327.15), IL-10 (25–5079.9), MCP-1 (31–5524.18), IFN-γ (4.7–4936.75), TNF-α (6.3–5164.32), and IL-12p70 (9.1–7128.78). According to the manufacturer’s instructions, where necessary, for sample intensities not within the theoretical limits of the standard curve, values could be extrapolated by applying a 5-parameter logistic curve fit option.

### 4.5. Spleen and Mesenteric Lymph Node Processing

Spleens and MLNs were processed for flow cytometry. Briefly, splenocyte and lymph node cell suspensions were obtained by gentle mechanical disruption with a potter homogeniser until complete disaggregation with cold PBS 1% EDTA. In the case of the spleen, erythrocytes were lysed by incubation with lysis buffer (Cytognos, Salamanca, Spain) for 15 min at room temperature.

Cells were stained with CD45, CD4, CD25, FoxP3, CD11b, Ly6G, and Ly6C mAbs, as indicated in the following section (Supplementary Table S1). Cells were gated based on forward/side scatter characteristics. Treg lymphocyte populations were defined as CD45^+^CD4^+^CD25^+^FoxP3^+^ cells. Myeloid populations were gated within CD45^+^CD11b^+^ cells. Neutrophils were determined by Ly6G^hi^Ly6C^lo^ expression and monocyte/macrophage population within the Ly6G^−^ Ly6C^+^ population.

### 4.6. Flow Cytometry

Before staining with specific antibodies, cells were incubated at 4 °C for 5 min with FcR Blocking Reagent (Milteny Biotec, Bergisch Gladbach, Germany) to block non-specific binding. Then, cells were stained with specific monoclonal antibodies (Supplementary Table S1) conjugated with different fluorochromes (Alexa Fluor 488, FITC, PE, PerCP, PECy5, Alexa Fluor 647 or APC) diluted in PBS (phosphate-buffered saline, Corning) 1% EDTA.

For the intracellular detection of Foxp3, cells were treated with a FACS permeabilization solution according to the manufacturer’s instructions (BD Biosciences). Analysis was performed on a FACSCalibur flow cytometer (BD Biosciences) (Centro de Citometría y Microscopía de Fluorescencia, Complutense University of Madrid) using FCS Express V3 software.

### 4.7. Splenocyte and MLN Cell Proliferation Assay

Cell suspensions from the spleens and MLNs were labelled with 2.5 μM of CFSE (Biolegend, San Diego, CA, USA), following the manufacturer’s instructions, and stimulated with concavalin A (ConA, Sigma-Aldrich, St Louis, MO,USA) (3 μg/mL) and phorbol 12-myristate 13-acetate (PMA, Sigma-Aldrich) (10 ng/mL). Four days after stimulation, splenocyte proliferation in the CD3^+^population was determined by flow cytometry using the CFSE dilution method. Treg lymphocyte populations (CD4^+^CD25^+^FoxP3^+^) were also analysed after four days of stimulation in the spleen and MLN cell suspensions.

### 4.8. Human PBMC and Monocyte Isolation

Human peripheral blood mononuclear cells (PBMCs) were obtained by density gradient centrifugation using lymphocyte isolation solution (Rafer, Zaragoza, Spain) from buffy coats of healthy volunteer donors (Centro de Transfusión de la Comunidad de Madrid, Spain). Monocytes were obtained from PBMCs by immunomagnetic isolation using anti-CD14 microbeads and a VarioMACS cell separator (Miltenyi Biotec, Bergisch Gladbach, Germany), following the manufacturer’s protocol.

### 4.9. MV130 Uptake Assay

MV130 bacteria were stained with 2.5 μM of CFSE (Biolegend), following the manufacturer’s instructions, to monitor their presence by flow cytometry. Human PBMCs were cultured with MV130-CFSE or MV130 as a negative control for 24 h. The uptake of MV130, with or without CFSE, by lymphocytes, monocytes, and granulocytes was analysed by flow cytometry, with populations defined by forward scatter (FSC) and side scatter (SSC) parameters.

### 4.10. Human Monocyte-Derived Macrophage Cultures

Human monocytes, isolated as previously described, were cultured in RPMI 1640 medium (Lonza, Basel, Switzerland) supplemented with 10% foetal bovine serum (Gibco, Thermo Fisher Scientific, Waltham, MA, USA), 100 U/mL penicillin, 100 μg/mL streptomycin, 2 mM L-glutamine, and 1 mM pyruvate (all from Lonza) in the presence of GM-CSF (5 ng/mL) to induce M1 macrophages or in the presence of M-CSF (10 ng/mL; both from Gibco; Thermo Fisher Scientific) to induce M2 macrophages. MV130 treatment (10^7^ bacteria/mL) was added at the beginning of the culture. After 3 days, additional 5 ng/mL GM-CSF or 10 ng/mL M-CSF was added to the corresponding macrophage cultures. On day 6 of the culture, macrophage phenotype was analysed by flow cytometry using CD14 (Immunostep, Salamanca, Spain) and CD163 (Biolegend) (Supplementary Table S1). The percentage of viable cells was analysed by flow cytometry using annexin V-FITC and propidium iodide (both from Biolegend).

Supernatants from the cultures were harvested and cytokine secretion was determined by LegendPlex Human Essential Immune Response (Biolegend), according to the manufacturer’s instructions. The detection ranges were as follows (pg/mL): IL-4 (0.67–11837.9), CXCL10 (1.55–5401.68), TNF-α and IL-10 (1.45–8131.49).

### 4.11. RNA Extraction and Gene Expression Analysis by qRT-PCR

Cells were lysed to perform RNA purification using the SPEEDTOOLS Total RNA Extraction kit (Biotools, Madrid, Spain) following the manufacturer’s protocol. A high-capacity cDNA Reverse transcription kit (Applied Biosystems; Thermo Fisher Scientific) was used for the synthesis of the cDNA, following the manufacturer’s instructions. Quantitative real-time PCR (qRT-PCR) was performed using specific predesigned TaqMan Gene expression assays for different genes (Applied Biosystems; Thermo Fisher Scientific) (Supplemental Table S2). All PCR reactions were set in duplicates using the TaqMan Gene Expression Master Mix (Applied Biosystems; Thermo Fisher Scientific). The amplification and detection were performed using a 7.900HT Fast Real-time PCR System (Centro de Genómica, Complutense University of Madrid). The ΔCT method was employed, using GNB2L1 as a reference gene to normalise gene expression.

### 4.12. Tissue Immunofluorescence

For immunofluorescence studies, the last part of the distal third of the colon from each animal was embedded in an OCT compound (Thermo Fisher Scientific, Waltham, MA, USA), frozen, and stored at −80 °C until processing. Colon sections (5 µm thick) were first blocked with 5% normal donkey serum in PBS (phosphate-buffered saline, Corning) and subsequently incubated with the following primary antibodies or isotype control in 10% bovine serum albumin (BSA) in PBS for one hour at room temperature. We used anti-CD45 Alexa Fluor™ 488 and anti-CD68 Alexa Fluor™ 594 (BioLegen), anti-CD206 (Abcam, Cambridge, UK;), and anti-CD15 (Biolegend) as primary antibodies and Rat IgG2b κ, Rat IgG2a κ, Rabbit Polyclonal and Mouse IgM, and κ Isotype Control Antibodies (Biolegend), respectively, as the staining controls. Sections stained with the anti-CD206 or anti-CD15 primary antibodies were incubated with rabbit IgG and mouse IgM secondary Alexa Fluor™ 594 antibodies (Invitrogen Life Technologies Grand Island, NY, USA) for a period of 45 min. Nuclear counterstaining was performed using Hoechst 33342 (Invitrogen; Life Technologies Grand Island, NY, USA), and the sections were mounted with FluorSave (Millipore Burlington, MA, USA). The slides were then examined using a Nikon Eclipse Ci fluorescence microscope. Images were acquired using Nikon DS-U3 digital sight (Nikon, Tokyo, Japan) and Nis-Elements D viewer software (https://www.microscope.healthcare.nikon.com/products/software/nis-elements/software-resources, last access 2 October 2024), and finally, images were processed and measured using Fiji-win64 software (https://fiji.sc/, last access 2 October 2024).

### 4.13. Tissue Myeloperoxidase (MPO) Activity

The proximal third of the colonic tissue was obtained from either the control or DSS-treated animals, rinsed with cold PBS, blotted dry, and immediately frozen in liquid nitrogen. Samples were stored at –80 °C until being thawed for the determination of myeloperoxidase activity using the O-dianisidine method. Briefly, colon samples were homogenised at 25 mg/mL in phosphate buffer (20 mM, pH 6.0) containing 0.5% hexadecyltrimethylamonium bromide (HTAB) (Thermo Fisher Scientific). The samples were centrifuged at 12,000 rpm for 15 min at 4 °C. The supernatants were diluted 1/30 with 20 mM phosphate buffer (pH 6.0) containing 0.04 mg/mL O-dianisidine (Sigma-Aldrich) and 0.002% H_2_O_2_ (*v*/*v*). Changes in absorbance at 450 nm were recorded with a spectrophotometer every 1 min for 30 min. MPO activity was expressed as units per mg of wet tissue.

### 4.14. Measurement of Colonic Protein

The middle third of the colon tissue was rinsed with cold PBS, blotted dry, and immediately frozen in liquid nitrogen. Samples were stored at –80 °C until being thawed for the detection of different inflammatory proteins. Briefly, colon samples were weighed and homogenised with an appropriate volume of protein extraction buffer containing lysis buffer and protease inhibitor (Merck, KGaA, Darmstadt, Germany) according to their weight (300 μL approx.). After homogenisation, samples were centrifuged at 5000 rpm for 5 min and the supernatant was used for the measurement of a variety of proteins using the Legendplex Mouse Inflammation panel (Biolegend), according to the manufacturer’s instructions. The detection ranges were as follows (pg/mL): IL-1α (0.52–19,135.38), IL-1β (2.2–7674.61), IL-6 (7.31–10,548.62), CCL2 (6.15–11,168.43), IFN-β (2.2–7674.61).

### 4.15. Statistical Analysis

All data are presented as mean ± SEM of the specified parameters and were analysed using GraphPad Prism version 8.0.2 software (GraphPad Inc, San Diego, CA, USA). The Shapiro–Wilk test was used to assess the normality of the variables. Statistical comparisons between two experimental groups were performed using the parametric Student’s *t*-test. Multiple group comparisons were performed using a one-way analysis of variance (ANOVA) for parametric variables or Kruskal–Wallis for non-parametric variables, as appropriate. *p* ≤ 0.05 (*), *p* ≤ 0.01 (**), *p* ≤ 0.001 (***), and *p* ≤ 0.0001 (****) values were considered statistically significant.

## Data Availability

All data generated and/or analysed during this study are included in this published article and its additional files. All the data are available on request from the corresponding author.

## Supplementary Materials

The following supporting information can be downloaded at: https://www.mdpi.com/article/10.3390/ijms252413629/s1.
