# Supplementary material for: Mucosal Bacterial Immunotherapy Attenuates the Development of Experimental Colitis by Reducing Inflammation Through the Regulation of Myeloid Cells
Source: Int J Mol Sci. 2024 Dec 20;25(24):13629. doi: 10.3390/ijms252413629 (PMC11728189; doi:10.3390/ijms252413629)
Supplement: Supplementary file 1 [file ijms-25-13629-s001.zip › ijms-3343726-supplementary.pdf]

Supplementary Figure S1

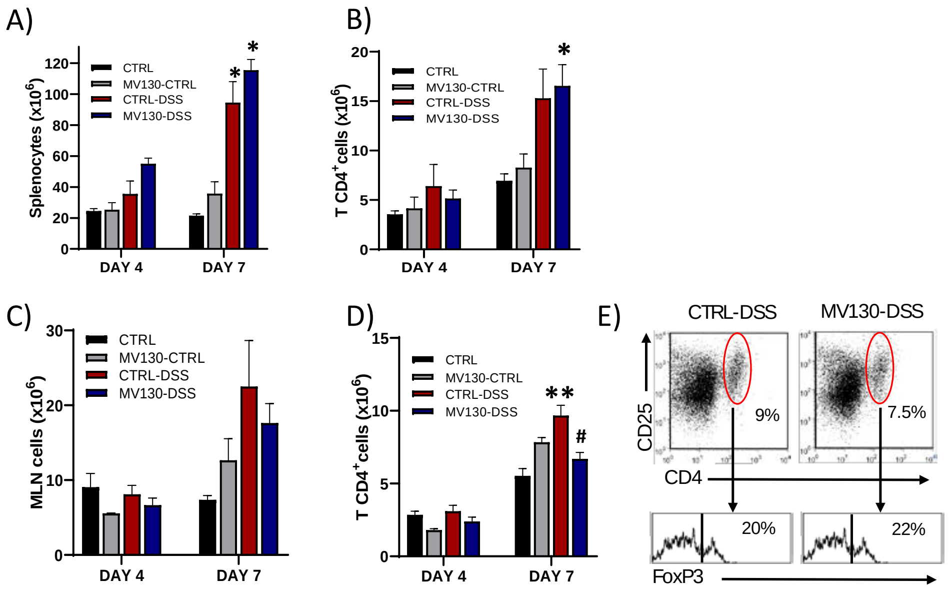

Figure S1. MV130 effect in spleen and mesenteric lymph node during acute colitis. Mice were treated as described in figure 1A, and spleen and mesenteric lymph nodes were collected on day 4 and 7 after colitis induction. (**A-B**) Absolute numbers of total splenocytes and T CD4$^+$ lymphocytes recovered from the spleen of mice on days 4 and 7 after colitis induction (n=3-4 per group). (**C-D**) Absolute numbers of total cells and T CD4$^+$ lymphocytes recovered from the MLNs of mice on days 4 and 7 after colitis induction (n=3-4 per group). (**E**) Flow cytometry analysis of the Treg lymphocyte population (CD4$^+$CD25$^+$FoxP3$^+$) in MLN after four days of stimulation with ConA+PMA in CTRL-DSS and MV130-DSS mice. Representative dot plots with the percentage of CD4$^+$CD25$^+$FoxP3$^+$ cell was shown (n=4 per group). Results represent the mean ± SEM of three-four animals per group and the significance is indicated with respect to CTRL (*) or CTRL-DSS (#). *p<0.05; **p<0.01 by Kruskal-Wallis test.

Supplemental Table S1. Human and mouse antibodies used for flow cytometry and immunostaining

Human Target Molecule	Clone	Dilution	Manufacturer
CD163	GH1/G1	3/20	Biolegend
CD3	SK7	2/20	
CD14	47-3D6	3/20	Inmunostep
**Mouse Target Molecule**	**Clone**	**Dilution**	**Manufacturer**
CD45	30-F11	0,5/20	Biolegend
CD3	17A2	2/20	
CD4	GK1.5	1,25/20	
CD25	PC61	1,25/20	
CD11b	M1/70	0,5/20	
Ly6G	HK1.4	1/20	
Ly6C	1A8	1/20	
CD68	FA-11	1/100	
CD15	MC-480	1/50	
CD206	polyclonal	1/100	Abcam
FoxP3	FJK-16s	5/20	Thermo Fisher Scientific
**Isotype Control**	**Clone**	**Dilution**	**Manufacturer**
Rat IgG2a, κ	RTK2758	1/100	Biolegend
Rabbit Polyclonal	Poly29108	1/100	
Mouse IgM, κ	MM-30	1/100	
Rat IgG2b, κ	RTK4530	1/100	

Supplemental Table S2: TaqMan Gene expression assay

**Gene**	**Reference**
GNB2L1	Hs00272002_m1
IL-10	Hs00961622_m1
TNFa	Hs00174128_m1
CCL2	Hs00234140_m1
VEGF	Hs00900055_m1
IDO1	Hs00984148_m1
